# Influence of Heat Stress on Body Surface Temperature and Blood Metabolic, Endocrine, and Inflammatory Parameters and Their Correlation in Cows

**DOI:** 10.3390/metabo14020104

**Published:** 2024-02-02

**Authors:** Bojan Blond, Mira Majkić, Jovan Spasojević, Slavča Hristov, Miodrag Radinović, Sandra Nikolić, Ljiljana Anđušić, Aleksandar Čukić, Maja Došenović Marinković, Biljana Delić Vujanović, Nemanja Obradović, Marko Cincović

**Affiliations:** 1 Department of Veterinary Medicine, Faculty of Agriculture, University of Novi Sad, Square Dositeja Obradovića 7, 21000 Novi Sad, Serbia; bojan.blond@gmail.com (B.B.); mira.majkic@polj.edu.rs (M.M.); jovan.spasojevic@polj.edu.rs (J.S.); miodrag.radinovic@polj.edu.rs (M.R.); sandra.nikolic@polj.edu.rs (S.N.); 2 Faculty of Agriculture, University of Belgrade, Nemanjina 6, Zemun, 11080 Belgrade, Serbia; hristovs@agrif.bg.ac.rs; 3 Faculty of Agriculture, University of Priština in Kosovska Mitrovica, Kopaonička bb, 38219 Lešak, Serbia; ljiljana.andjusic@pr.ac.rs (L.A.); aleksandar.cukic@pr.ac.rs (A.Č.); 4 Academy of Applied Studies Šabac, Dobropoljska 5, 15000 Šabac, Serbia; m.d.marinkovic@akademijasabac.edu.rs (M.D.M.); b.d.vujanovic@akademijasabac.edu.rs (B.D.V.); 5 Pasteur Institute Novi Sad, Hajduk Veljkova 1, 21000 Novi Sad, Serbia; nemanjanskonzole@gmail.com

**Keywords:** heat stress, cow, metabolic adaptation, infrared thermography, correlation

## Abstract

This study aimed to determine whether heat stress affected the values and correlations of metabolic, endocrinological, and inflammatory parameters as well as the rectal and body surface temperature of cows in the early and middle stages of lactation. This experiment was conducted in May (thermoneutral period), June (mild heat stress), and July (moderate to severe heat stress). In each period we included 15 cows in early lactation and 15 in mid-lactation. The increase in rectal and body surface temperatures (°C) in moderate to severe heat stress compared to the thermoneutral period in different regions was significant (*p* < 0.01) and the results are presented as mean and [95%CI]: rectal + 0.9 [0.81–1.02], eye + 6 [5.74–6.25], ear + 13 [11.9–14.0], nose + 3.5 [3.22–3.71], forehead + 6.6 [6.43–6.75], whole head + 7.5 [7.36–7.68], abdomen + 8.5 [8.25–8.77], udder + 7.5 [7.38–7.65], front limb + 6 [5.89–6.12], hind limb + 3.6 [3.46–3.72], and whole body + 9 [8.80–9.21]. During heat stress (in both mild and moderate to severe stress compared to a thermoneutral period), an increase in the values of extracellular heat shock protein 70 (eHsp70), tumor necrosis factor α (TNFα), cortisol (CORT), insulin (INS), revised quantitative insulin sensitivity check index (RQUICKI), urea, creatinine, total bilirubin, aspartate transpaminase (AST), gamma-glutamyl transferase (GGT), lactate dehydrogenase (LDH), and creatin kinase (CK) occurred, as well as a decrease in the values of triiodothyronine (T3), thyroxine (T4), non-esterified fatty acids (NEFA), glucose (GLU), β-Hydroxybutyrate (BHB), calcium, phosphorus, total protein (TPROT), albumin (ALB), triglycerides (TGCs), and cholesterol (CHOL). In cows in early lactation compared to cows in mid-lactation, there was a significantly larger increase (*p* < 0.01) in the values of eHsp70, TNFα, GLU, RQUICKI, and GGT, while the INS increase was smaller during the three experimental periods. The decrease in the values of Ca, CHOL, and TGC was more pronounced in cows in early lactation compared to cows in mid-lactation during the three experimental periods. Rectal temperature was related to eHsp70 (r = 0.38, *p* < 0.001) and TNFα (r = 0.36, *p* < 0.01) and showed non-significant poor correlations with other blood parameters. Blood parameters correlate with body surface temperature, with the following most common results: eHsp70 and TNFα showed a moderately to strongly significant positive correlation (r = 0.79–0.96, *p* < 0.001); CORT, INS, and Creat showed fairly to moderately significant positive correlations; T3, T4, NEFA and GLU showed fairly to moderately significant negative correlations (r = 0.3–0.79; *p* < 0.01); RQUICKI, urea, AST, and GGT showed fairly and significantly positive correlations; and TGC, CHOL, TPROT, and ALB showed fairly and significantly negative correlations (r = 0.3–0.59; *p* < 0.01). Measuring the surface temperature of the whole body or head can be a useful tool in evaluating the metabolic response of cows because it has demonstrated an association with inflammation (TNFα, eHsp70), endocrine response (CORT, T3, T4), the increased use of glucose and decreased use of lipids for energy purposes (INS, NEFA, GLU, and RQUICKI), and protein catabolism (ALB, TPROT, urea, Creat), which underlies thermolysis and thermogenesis in cows under heat stress. In future research, it is necessary to examine the causality between body surface area and metabolic parameters.

## 1. Introduction

There have been favorable conditions for the development of heat stress in cows over the past 20 years in the region of Vojvodina (Serbia). The increase in air temperature that accompanies global warming, in combination with the existing values of air humidity, creates a temperature–humidity index (THI) typical of heat stress (THI > 72) [1]. The maximal THI index value has been increasing over the past 10 years by more than 4 units in the summer months, suggesting that cows in this region are becoming more and more susceptible to heat stress [1]. Heat waves are becoming more frequent and intense in this region, and there are more tropical days and nights overall. As a result, summertime maximum daily temperatures frequently rise to over 35 or 40 °C [2,3]. Cows are homeothermic animals with a body temperature in the range of 38.5–39.5 °C and a thermoneutral zone of −1.1 to +25 °C [4]. When cows are exposed to temperatures that exceed the upper limit of the thermoneutral zone, a number of physiological responses occur, including an increase in body temperature, and productive metabolic, endocrine, and inflammatory responses [5,6].

The rectal temperature of cows and the values of the THI index positively correlate, which indicates that heat stress will cause an increase in body temperature (38.5 °C when THI < 68, compared to 39.2° when THI > 80) [7]. Body temperature is measured using a rectal thermometer, and the connection between rectal temperature and core body temperature makes this method very suitable for assessing the body's response to heat stress [8]. However, rectal thermometry requires the capture, immobilization, and additional manipulation of cows, so, to avoid additional stress, methods for non-invasive and non-contact thermometry have been devised [9]. Due to high temperature, vasodilatation, and heat loss, i.e., an increase in thermal radiation taking place, which we record with a thermal camera, the most sensitive places where these changes can be measured are called thermal windows [10]. These are the areas close to the skin’s surface that are not covered in hair and contain rich vascular tangles, a dense network of capillaries, and anastomoses [10]. In cows experiencing heat stress, heat loss through cutaneous evaporation is the most effective for maintaining homeothermy [11]. This process involves the entire body surface, so a thermal camera can be used to record the increase in the temperature of the skin surface of all anatomical regions [12].

The metabolic and productive response of cows to heat stress occurs either as a consequence of the direct effect of high temperatures and the development of hyperthermia or as a consequence of metabolic rearrangement aimed at reducing the heat production of the metabolic processes of the organism [13]. As a result of the increased ambient temperature, food intake is reduced in order to avoid its caloric effect and postprandial warming of the body. Reduced food intake leads to a decrease in milk production, which reduces the sensitivity of cows to heat stress [14]. Metabolic processes gravitate toward the lower-calorie-producing reactions, so that cows change fuel during heat stress [13,15]. During heat stress, the production of glucose in the liver increases, and glucose is directed towards the muscles to be used for the body’s energy purposes, and not towards the udder for milk production. In parallel with the increase in glucose, the concentration of insulin also increases. Insulin enables the increased use of glucose in muscle tissues for energy needs. Insulin simultaneously exerts an antilipolytic effect and reduces lipolysis and the use of fatty acids for energy purposes. Burning one gram of glucose gives much less energy than burning one gram of fat, so choosing glucose as a fuel reduces the caloric effect of metabolic reactions. Proteolysis and the use of glucogenic amino acids as a source of glucose also occur in heat stress. Elevated ambient temperature directly increases the activity of the adrenal gland and cortisol, and the activity of T3 and T4 decreases due to their thermogenic effect [16]. During heat stress, certain changes occur that can increase body temperature. Heat stress directly increases Hsp70 levels in cells, which protects them from stress, but if it is found extracellularly it exacerbates the inflammatory response [17,18]. Inflammation can occur as a result of the penetration of fecal metabolites from the digestive organs [19]. 

The lactation period significantly affects the values of the metabolic parameters in cows [20]. Season and lactation stage had significant effects on the body temperature rhythm in cows [21]. The stress caused by the onset of lactation and heat stress has significant similarities due to the development of a negative energy balance. Inflammatory and endocrine responses are similar for both stressors, while the metabolic response differs. Metabolic adaptation to heat stress is a special type of homeoretic adaptation that differs from homeoresis in early lactation. Thus, in cows in early lactation, increased lipolysis and insulin resistance occur in order to direct glucose to the udder, which supports lactation (homeoresis), and, in heat stress, sensitivity to insulin increases, lipolysis decreases, and glucose is redirected to the tissue [22]. Changes in metabolic processes can be accompanied by changes in the activity of certain enzymes that indicate the functional status of the liver and biliary tract (GGT, AST, ALP) and the muscular system (AST, LDH, CK) [6,20].

A significant correlation has been established between THI and the body surface temperature in cows, and it has been noted that body surface temperature varies due to a variety of metabolic and endocrine conditions [23,24]. Previous studies in sheep have demonstrated that the temperature of certain regions was significantly related to the THI index, but also to certain metabolic parameters [25]. This particular type of connection has not yet been investigated in dairy cows. This study aims to ascertain whether heat stress affects the values of metabolic, endocrinological, and inflammatory parameters as well as the body surface temperature in cows in the early and middle stages of lactation. Additionally, it seeks to determine whether there is a relationship between the surface temperature of various body regions and metabolic parameters.

## 2. Materials and Methods

### 2.1. Animals and Management 

The experiment was conducted in Serbia, Vojvodina region, over three periods: May (thermoneutral period), June (mild heat stress), and July (moderate to severe heat stress) 2023. A total of 90 cows (thirty in each period) of the Holstein-Friesian breed were included in the experiment. In each period we grouped one half of cows in the first month of lactation (early lactation) with a second half in the fourth and fifth month of lactation (mid-lactation). The experiment included healthy cows without visible pathological changes or deposits of dirt on their skin and mucous membranes or visible signs of estrus (which can affect infrared thermography). The cows were in their second and third lactation and 3–5 years old. All cows had birthed one healthy calf, and cows with problems during parturition were not included in the experiment. Cows were kept in the standard free stalls system of cow housing. The building was completely covered with a roof and the animals were in the shade. Water was available ad libitum. Cows were fed twice daily using a TMR mixture according to standards [26]. Components of the cows’ meal (in kg of dry matter) included corn silage—multiple grains 8.2; haylage of alfalfa 1.65; hay of alfalfa 1.94; silane beet noodles 1.8; beer trope fresh 0.96; corn grain 5.38; barley grain 0.61; oil shot rapeseed 0.92; soybean meal 0.87; sunflower meal 1.44; extruded flaxseed 0.55; livestock chalk 0.13; livestock salt 0.06; 14-Baking soda 0.06; MgO 0.05; premix 0.18; Phosphozel 0.12; Zenural (urea) 0.18; Bentonite (Mycotoxin adsorbent) 0.02; dairy fat c16 0.39. Chemical composition included (%) dry matter 52.89; crude protein 17.01; crude fat 4.76; raw ash 6.81; NDF 28.13; ADF 16.53; lignin 3.1; Ca 0.97; P 0.45; dry matter 25.3 kg; NEL 7.24 MJ/kg. TMR is similar in its selection of nutrients, but the balancing of nutrients in the ration is carried out to meet the nutritional levels characteristic of each lactation period and current milk production. In early lactation, compared to mid-lactation, we have crude protein 16–17:17–17.5% DM, soluble protein 30–34:32–36%, crude protein, Forage NDF, 19–25:25–26% DM, and Approximate concentrate, 50–60:40–50% DM. When feeding total mixed rations differences were avoided between rations that exceed 10 to 15% for milking groups to avoid excessive drops in production when moving to a lower group. Concentrate level is dependent on forage quality, amounts and types of byproduct feeds, and energy-corrected milk.

### 2.2. Ambient Temperature–Humidity Index and Periods of the Experiment

In order to determine the temperature–humidity index (THI), temperature and air humidity were measured twice a day during the three different periods of the experiment, at 05:00–07:00 (THI morning) and 13:00–15:00 (THI day). The temperature–humidity index was calculated using the standard formula [27], according to the data from the Republic Hydrometeorological Service of Serbia. Standard automatic weather stations are used to measure temperature and air humidity. These stations measure air temperature, air humidity, air pressure, wind direction, and speed. The temperature is measured in a prescribed meteorological shelter. The data are visible on the Institute’s website in almost real time (~20 min interval), and the data have been analyzed in detail from the available daily and monthly bulletins.

Measurement of temperature and humidity and determination of the THI index were performed daily. Based on the value of the THI index during the day, three groups of animals were formed: cows in a thermoneutral period (THI < 72), cows in mild heat stress (THI = 70–80), and cows in moderate to severe heat stress (THI > 80). The mentioned daily values of the THI index had to be determined during 10 consecutive days from 13:00–15:00 h, in order to measure body surface temperature and perform blood analysis on the last day of each period. 

### 2.3. Body Surface and Rectal Temperatures

The body surface temperature was measured in all animals by collecting images of different parts of their body's thermal window: eye temperature (EYT), ear temperature (EAT), nose temperature (NT), forehead temperature (FHT), whole head temperature (WHT), abdomen temperature (AT), front limb temperature (FLT), hind limb temperature (HLT), udder temperature (UT), and whole body temperature (WBT). Thermographic images were taken with an infrared camera, Testo 865 (Germany). Thermograms with an emissivity coefficient of 0.95 were recorded from each cow. Thermal images were obtained from a distance of approximately 1–2 m. Thermal imaging was performed in a time interval from 13:00–15:00 h on the last day of each period. Each photo was taken in triplicate, taking up to 1 min per animal. All recordings were made at the same distance and at the same angle, without any additional disturbance to the cow. In order to avoid the variability of the measured temperature that can occur at certain points due to measurement uncertainty or other factors, we opted for much higher-quality and less variable data—the average temperature of the selected surface of the cow’s body. Insignificant deviations that were at the level of up to 0.05 °C for a very small number of points have no influence on the average value or on the shape of the frequency distribution of temperature and color on the thermogram. The main findings during thermography are graphically presented as thermographs and frequency distributions of each surface temperature. Rectal temperature was measured with a standard digital thermometer (Kruuse). 

### 2.4. Blood Sampling and Metabolic Parameters Analysis

Blood sampling was performed immediately after infrared thermography via jugular venipuncture using 10 mL serum separation tubes. During blood sampling, the existing passage corridor, to which the cows were accustomed, was used and standard head fixation was performed. The blood was sampled in just a few minutes and in sufficient volume, so the effect of pre-analytical factors was minimal. In order to separate the serum better, it was additionally centrifuged for 5 min at 3000 g. The serum samples were then collected and placed in vials, frozen at −20 °C, and transported in laboratory refrigerators to the Laboratory of Pathophysiology, Department of Veterinary Medicine, University of Novi Sad. We performed analysis of the following biochemical parameters: cortisol (CORT), triiodothyronine (T3), thyroxine (T4), insulin (INS), non-esterified fatty acids (NEFA), beta-hydroxybutyrate (BHB), glucose (GLU), calcium (Ca), inorganic phosphates (Ps), total protein (TPROT), albumin (ALB), urea, triglycerides (TGCs), cholesterol (CHOL), total bilirubin (TBIL), creatinine (CR), aspartate aminotransferase (AST), gamma-glutamyl transferase (GGT), lactate dehydrogenase (LDH), alkaline phosphatase (ALP), and creatine kinase (CK). Standard kits from Randox (Randox, London, UK), for NEFA, and BioSystem (Barcelona, Spain), for the other parameters, were used with a Rayto Chemray 120 spectrophotometer (Rayto Life and Analytical Sciences, Shenzhen, China). An automated immunoassay analyzer TOSOH AIA-360 (Tosoh Bioscience, Tokyo, Japan) was used for endocrinological analyses. For the estimation of insulin sensitivity we used a revised quantitative insulin sensitivity check index and formula: RQUICKI = 1/[log (glucose mg/dL) + log (insulin μU/mL) + log (NEFA mmol/L)] [28]. TNF-α was measured using standard kit manufactured by Cloud-Clone Corp (Wuhan, China; Intra-Assay: CV < 10%; Inter-Assay: CV < 12%; assay sensitivity 3.1 pg/mL) and a Fluoroscan Ascent FL reader (Thermo Scientific, Waltham, MA, USA). Concentration of serum eHsp70 was determined via an ELISA colorimetric kit (Cusabio, Wuhan, China; Intra-Assay: CV < 15%; Inter-Assay: CV < 15%; assay sensitivity 1.25 ng/mL) using ELISA reader and washer by Rayto (Shenzhen, China).

### 2.5. Statistical Analysis

Statistical analysis included the use of GLM and ANOVA analyses to examine the effect of heat stress intensity, lactation period, and heat stress × lactation period interactions on the value of body surface temperature measured by the thermography of different body parts and blood biochemical parameters. The influence of heat stress is a fixed factor and is represented by the value of the THI index through three categorical variables: no heat stress—thermoneutral period (THI < 72), mild heat stress (THI = 72–80), and moderate to severe heat stress (THI > 80). The second factor is the period of lactation, which is also a fixed factor with two categorical variables: cows in early lactation (first month of lactation) and cows in mid-lactation (fourth and fifth month of lactation). The linear mathematical model for a two-factor experiment is Yijk = m + ai + bj + (ab)ij + eijk. This model expresses the value of the response variable, Y, as the sum of five components: m—the mean; ai—the contribution of the *i*-th level of factor A; bj—the contribution of the j-th level of factor B; (ab)ij—the combined contribution of the *i*-th level of factor A and the *j*-th level of factor B; eijk—the contribution of the *k*-th individual (called error). The assumptions of general linear model were checked in SPSS, including the continuous dependent variable, categorical independent variable with two or more related groups, normal data distribution, significant outliers, and equal variances of the differences between all combinations of related groups. The relationship between body surface temperature and the blood parameters was examined using the Pearson correlation coefficient. We evaluated the strength of correlations and their statistical significance. The strength of the correlation was assessed based on the correlation coefficient as either no correlation (N, r = 0.00–0.09), poor (P, r = 0.1–0.29), fair (F, r = 0.3–0.59), moderate (M, r = 0.6–0.79), strong (S, r = 0.8–0.99), or perfect (Pe, r = 1). Statistical significance was assessed as significant (*p* < 0.05), highly significant (*p* < 0.01), or very highly significant (*p* < 0.001). SPSS statistics software (IBM, Armonk, NY, USA) was used. All statistical tests were considered significant if *p* < 0.05. 

## 3. Results

**Figure 1:**
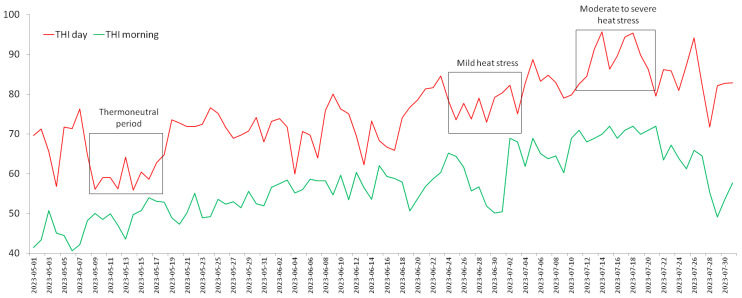
Temperature–humidity index (THI) during experimental period (May–July 2023).

The value of the THI index was in the thermoneutral zone during the night measurements. During the day, the THI index was in the thermoneutral zone in May, while in June and July the value of the THI index was in the heat stress zone. Heat stress was mild in the last week of June, while in July the THI value indicated moderate to severe heat stress. The results are presented in Figure 1.

**Figure 2:**
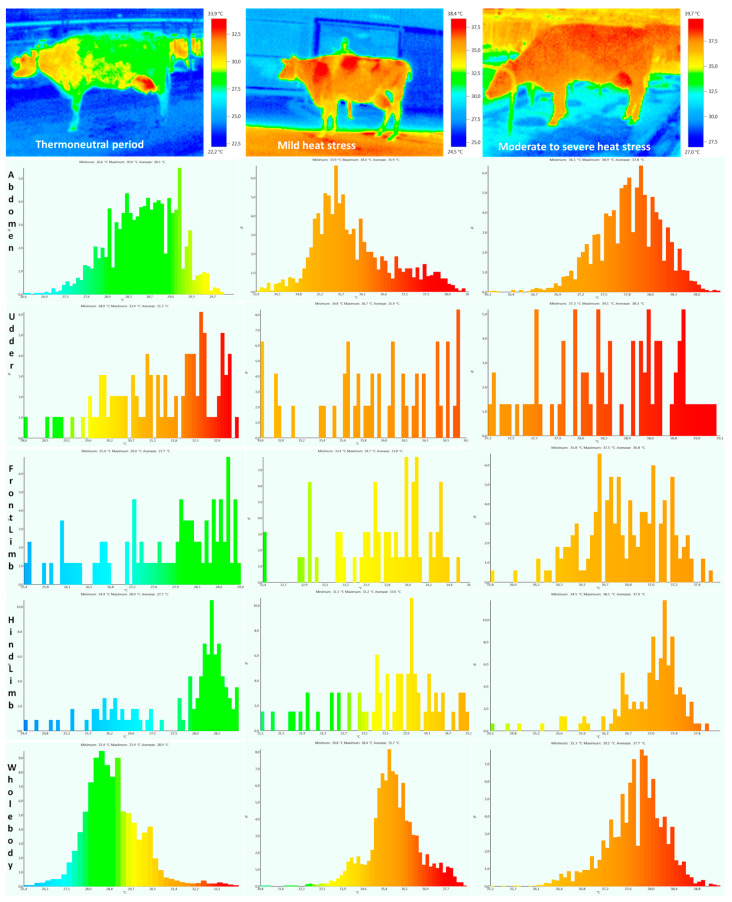
Infrared thermograms and frequency distribution of body surface temperatures of a cow (left lateral view).

**Figure 3:**
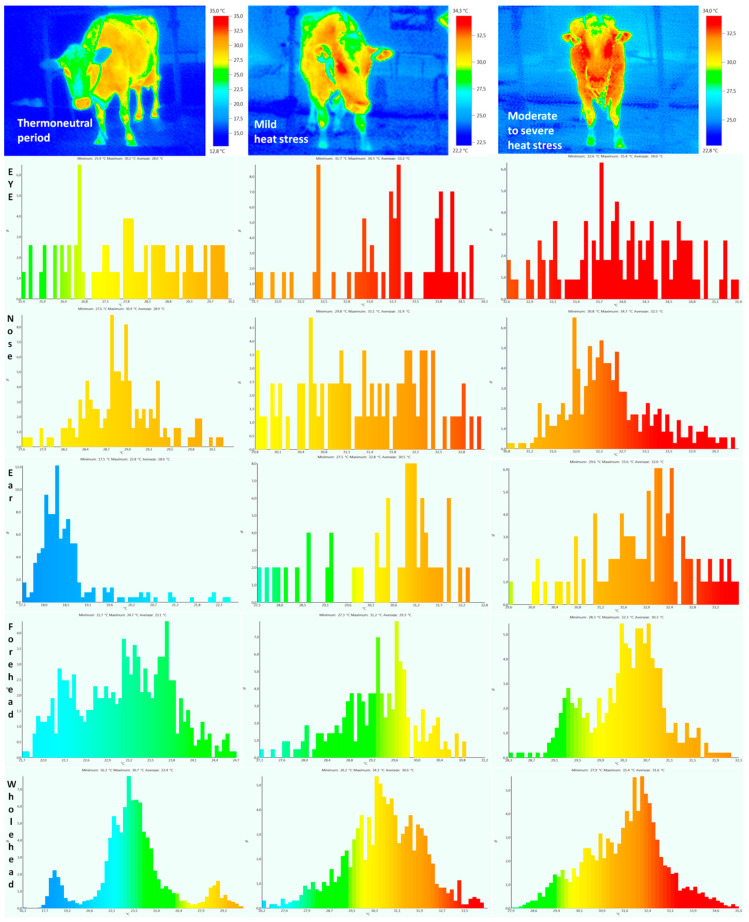
Infrared thermograms and frequency distribution of body surface temperatures of cow head.

Body surface temperature was significantly higher in moderate stress compared to the thermoneutral period, as well as in moderate to severe heat stress compared to mild heat stress, so the impact of stress was highly significant (*p* < 0.01) or significant (for rectal temperature *p* < 0.05) (Table 1). Typical thermograms and the temperature frequency distribution of different body surfaces over all three experimental periods are presented in Figure 2 and Figure 3. The increase in temperature in mild heat stress compared to the thermoneutral period was as follows: eye +5 °C [95%CI, 4.86–5.14], ear +11 °C [95%CI, 10.81–11.22], nose +2 °C [95%CI, 1.89–2.11], forehead +6 °C [95%CI, 5.73–6.29], whole head +7 °C [95%CI, 6.86–7.16], abdomen +7 °C [95%CI, 6.85–7.20], udder +5 °C [95%CI, 4.83–5.19], front limb +5 °C [95%CI, 4.9–5.11], hind limb +6 °C [95%CI, 5.89–6.13], and whole body +7 °C [95%CI, 6.65–7.39]. The temperature increase in moderate to severe stress compared to mild heat stress was as follows: eye +1 °C [95%CI, 0.92–1.12], ear +2 °C [95%CI, 1.84–2.13], nose +0.5 °C [95%CI, 0.46–0.54], forehead +0.6 °C [95%CI, 0.56–0.63], whole head +0.5 °C [95%CI, 0.48–0.52], abdomen +1.5 °C [95%CI, 1.38–1.61], udder +2.5 °C [95%CI, 2.32–2.62], front limb +3 °C [95%CI, 2.85–3.16], hind limb +1.8 °C [95%CI, 1.72–1.89], and whole body +2 °C [95%CI, 1.81–2.20]. The increase in body surface temperature in moderate to severe heat stress compared to the thermoneutral period in different regions was as follows: eye +6 °C [95%CI, 5.74–6.25], ear +13 °C [95%CI, 11.9–14.0], nose +3.5 °C [95%CI, 3.22–3.71], forehead +6.6 °C [95%CI, 6.43–6.75], whole head +7.5 °C [95%CI, 7.36–7.68], abdomen +8.5 °C [95%CI, 8.25–8.77], udder +7.5 °C [95%CI, 7.38–7.65], front limb +6 °C [95%CI, 5.89–6.12], hind limb +3.6 °C [95%CI, 3.46–3.72], and whole body +9 °C [95%CI, 8.80–9.21]. The period of lactation had no significant effect on the body surface temperature value (*p* > 0.05). No interaction between heat stress and the lactation period was noticed, so body temperature increased equally, due to heat stress, in cows in early and mid-lactation (*p* > 0.05).

The values of blood parameters and the impact of the investigated factors are presented in Table 2. Heat stress shows a significant influence on the value of metabolic parameters at a statistically significant (*p* < 0.05) or highly significant level (*p* < 0.01). During heat stress, an increase in the values of eHsp70 (*p* = 0.001), TNFα (*p* = 0.001), CORT (*p* = 0.001), INS (*p* = 0.009), RQUICKI (*p* = 0.001), urea (*p* = 0.045), Creat (*p* = 0.045), TBIL (*p* = 0.009), AST (*p* = 0.001), GGT (*p* = 0.001), LDH, and CK occurred, as well as a decrease in the values of T3 (*p* = 0.001), T4 (*p* = 0.001), NEFA (*p* = 0.001), GLU (*p* = 0.001), BHB (*p* = 0.008), Ca (*p* = 0.001), P (*p* = 0.001), TPROT (*p* = 0.001), ALB (*p* = 0.001), TGC (*p* = 0.007), and CHOL (*p* = 0.008). The value of ALP was not affected by any of the examined factors (*p* = 0.653). The lactation period had a significant influence, so significantly higher values of eHsp70 (*p* = 0.005), TNFα (*p* = 0.005), NEFA (*p* = 0.005), AST (*p* = 0.042), and GGT (*p* = 0.005) were noticed in cows in early lactation, as were lower values of INS (*p* = 0.048), RQUICKI (*p* = 0.012), Ca (*p* = 0.005), TGC (*p* = 0.043), and CHOL (*p* = 0.046). For various parameters, a significant heat stress × lactation period interaction was observed. In cows in early lactation, compared to cows in mid-lactation, there was a significantly larger increase in the value of eHsp70 (*p* = 0.005), TNFα (*p* = 0.005), GLU (*p* = 0.009), RQUICKI (*p* = 0.007), and GGT (*p* = 0.005), while the increase in INS was smaller (*p* = 0.046) during the three experimental periods. The decrease in the values of Ca (*p* = 0.005), CHOL (*p* = 0.045), and TGC (*p* = 0.048) was more pronounced in cows in early lactation compared to cows in mid-lactation during the three experimental periods.

The change in metabolite values depended on the intensity of the heat stress, with a statistically significant interaction between the heat stress period and lactation period (Table 2). The concentration of TNFα first decreased in mild heat stress and then increased significantly in moderate to severe heat stress in cows in mid-lactation. The concentration of CORT first increased in mild heat stress, but was slightly decreased in moderate to severe heat stress. In cows in early lactation, the value of INS first increased in mild heat stress and then decreased in moderate to severe heat stress, compared to mild heat stress. The concentrations of NEFA and ALB first decreased and then increased during the period of moderate to severe heat stress.

Correlation coefficients with a strong correlation and statistical significance are presented in Table 3. Rectal temperature showed a fair and significant positive correlation only with eHsp70 and TNFα, and non-significant poor correlations with other blood parameters. Blood parameters correlate with body surface temperature with the following most common results: eHsp70 and TNFα showed a moderately to strongly significant positive correlation (r = 0.79–0.96, *p* < 0.001); CORT, INS, and Creat showed fairly to moderately significant positive correlations; T3, T4, NEFA, and GLU showed fairly to moderately significant negative correlations (r = 0.3–0.79; *p* < 0.01); RQUICKI, urea, AST, and GGT showed fairly and significantly positive correlations; and TGC, CHOL, TPROT, and ALB showed fairly and significantly negative correlations (r = 0.3–0.59; *p* < 0.01); other parameters showed correlations of variable significance and strength.

The value of the correlation coefficients of blood parameters with the surface temperatures of different regions of the body can be very different, but it is always higher compared to the correlations with rectal temperature. The surface temperatures of different body areas showed different strengths of correlation with blood parameters, so that the strongest correlation was found between the temperature of the entire body, taken from the left lateral side; abdomen temperature; and the temperature of the entire surface of the head, taken from the front. Next in strength were correlations between the surface temperature of the udder, forehead, nose, and eye regions and blood parameters. The lowest linear correlations have been found between blood parameters and the temperature taken from the surface of the ear and front and hind limbs.

## 4. Discussion

The THI index in this research demonstrates that May is thermoneutral, and that cows experience moderate heat stress in June and moderate to severe heat stress in July. An increase in the value of the THI index from May to July is a common occurrence, and July is traditionally the warmest month in our region. The July of 2023 was the warmest month since temperature monitoring began, and it was the warmest month of the past 120,000 years according to various meteorological models. THI exhibits diurnal variation, so at night this index is in the thermoneutral zone, and during the day heat stress occurs. The arrival of the African heatwave from the Mediterranean region increased the number of tropical days and nights, so the cows experienced extreme heat stress because they did not have time to recover. In our experiment, we classified the intensity of heat stress as the thermoneutral period, mild heat stress (THI = 72–79), or moderate to severe heat stress (THI > 80). This classification is in accordance with previously recommended classifications [29]. As a threshold value for the occurrence of heat stress, we used THI = 72, which is a widespread and well-validated threshold value in various experimental studies [30,31]. This value is slightly lower in the Mediterranean region (THI = 68), and slightly higher in semi-humid and subtropical regions (THI = 78) [32]. Recent research in Germany demonstrates that at THI = 60 a decline in milk production occurs, and that this threshold also decreases for tropical regions [33,34]. THI limit values also depend on the formula used for calculation, but the dynamics of the changes in THI values during the year are the same regardless of the applied formula [35].

A positive correlation between the THI index and the measured temperature of different body areas was established in our experiment. A significant correlation was established between the THI value and body surface temperature, which was at the level of r = 0.58–0.81 (*p* < 0.001), but correlation was lower with the rectal temperature r = 0.29 (*p* < 0.05) (results not shown). Considering the non-contact temperature measurement, this can be carried out at one point or can examine a specific body area. It was concluded that using the average temperature of a particular body area rather than the single hottest spot is preferable based on the connection between the THI and the body surface temperature [36]. For this reason, we used the average temperature of a particular body part in our study. Measurements were made between 13:00 and 15:00 during the day, when heat stress was most noticeable. It was determined that obtaining measurements in the morning or evening would not allow us to predict the cows’ response to heat stress later in the day [37], indicating that the ideal time for infrared thermal imaging was selected.

The body surface temperature differed across the three experimental periods, such that the lowest temperature was established in the thermoneutral period, and the highest during moderate to severe heat stress (on average 11 °C higher than the thermoneutral period). The greatest difference was demonstrated between the thermoneutral period and mild stress, confirming that passing the THI = 72 threshold is important for the initiation of heat stress adaption. Infrared thermography represents a significant non-invasive method in the evaluation of the heat load of cows [38]. The average temperature of the entire head, measured frontally, was from 23.2 to 31.6 °C degrees, while the average temperature of the entire body, measured laterally, ranged from 28.8 to 37.7 °C. A temperature range of 28.4 to 36.6 °C was found by Salles et al. [39] when using data from a thermal imaging camera.

Several studies have demonstrated that the average temperature measured by infrared thermometry is lower than the rectal temperature by 2–7 °C [40], and the normal temperature of homeothermic cows is in the range of 38.0–39.3 °C [41]. Our temperatures are slightly lower compared to the values obtained so far, and the largest deviation occurs in the ear region. The reason is that in our experiment we measured the body surface temperatures of cows at a distance of 1 to 2 m, while in many other experiments the distance was much closer, from 0.2 to 1 m. In our study, we wanted to examine the possibility of a remote and completely non-invasive measurement of body temperature, without introducing animals into any enclosures. Distance and many other factors can affect the absolute value of the body surface temperature [42]. The temperature variation between different body regions ranged from 5 to 8.5 °C [39,43,44], which is consistent with our results, except for the ear region. The variability of temperature within the same region, expressed in standard deviations in earlier research, was from 0.44 to 3.24 [40,43,44]. In our study, this variability was from 0.32 to 1.21, and the most frequently measured SD was around 0.5. The obtained data indicate that we have achieved good measurement precision regardless of the slightly longer distance at which the animals were examined. The positive correlation between the body surface temperature of different regions coincides with previously obtained results [39], but our values are slightly higher because all cows from all experimental periods were included in the correlation analysis. The period of lactation did not show a statistically significant effect on body surface temperature, which is consistent with previous results [45]. It is known that the act of suckling or milking can affect the value of the infrared temperature of different regions of the body, and, in our experiment, we only observed cows that were in the period between two milkings and had no contact with calves [46].

Heat stress significantly affected the value of all investigated parameters except ALP. In cows under heat stress, the concentrations of eHsp70 and TNFα increase, indicating a systemic inflammatory response. The results of previous research have demonstrated an increase in the value of these two mediators together with other pro-inflammatory cytokines [47]. Since external temperatures are a significant stressor, the hypothalamic–pituitary–adrenal axis is activated with an increase in CORT values. CORT was considerably higher in cows exposed to heat stress. Cortisol concentration increased considerably at the moment of the first occurrence of heat stress, but in moderate to severe stress its increase was reduced. In acute heat stress, an increase in cortisol concentration was noticed, but its value decreased during the chronic course [48,49], which was compatible with our results. During induced heat stress, a decrease in the level of thyroid hormones occurs [50,51], which we also confirmed in our research. The decrease in the thyroid hormones’ level is more related to the change in ambient temperature than to the changes in the diet that occur during heat stress, so animals with a greater decrease in these hormones are more thermosensitive [52].

Changes in the metabolism of carbohydrates, fats, and proteins are very specific in cows during heat stress and are aimed at increasing the use of glucose for energy purposes and reducing the role of lipids, thereby reducing the thermal effects of metabolic processes. An increase in insulin values, a decrease in glucose and NEFA values with an increase in insulin sensitivity (RQUICKI index), and a decrease in BHB values were noticed. The decrease in the glucose value occurs as a result of reduced food intake, but also as a consequence of an altered post-absorptive phase of glucose metabolism, which results in increased insulin production and subsequently increased tissue glucose consumption [53]. The natural anabolic impact of insulin on a variety of bodily tissues, including fat tissue, may be the cause of increased insulin production. During heat stress, a decrease in NEFA and the utilization of lipids for energy purposes occurs, which reduces heat production [54]. These changes are the opposite of the metabolic processes in lactation, so, as a consequence of the aforementioned changes, there is a decrease in milk production during heat stress [55]. Changes in protein metabolism are reflected in the decreased total protein and albumin levels, along with an increase in urea and creatinine concentrations [56]. The obtained results support the hypothesis that heat stress causes cows to catabolize proteins, releasing amino acids that the body uses to convert glucose into energy [57,58]. Abbass et al. provided a detailed description of the interaction between glucose, insulin, and protein metabolism in heat stress, where the mentioned paradigms were confirmed [59].

In heat-stressed cows, the activity of liver enzymes increases and the concentrations of cholesterol and triglycerides decrease. This finding is specific for a functionally loaded liver during a negative energy balance, inflammation, and early lactation in cows [60]. An increase in the level of liver enzymes was noticed in small ruminants during heat stress [25], while in large ruminants the results were variable [61]. The concentrations of Ca and P were decreased, which coincides with previous results [61]. In dairy cows, P concentration decreases with the increase in glucose and insulin concentrations [62]. In cows in early lactation, significantly higher values of eHsp70, TNFα, NEFA, AST, and GGT are determined, as well as lower values of INS, RQUICKI, Ca, TGC, and CHOL compared to cows in mid-lactation, which is consistent with previous results and the presence of metabolic stress in early lactation [63,64,65]. Cows calved under heat stress exhibit lower values of NEFA, BHB, CHOL, and TGC during early lactation compared to cows calved in the thermoneutral period, which is confirmed by previous studies [66].

However, in cows in early lactation, the values of eHsp70, TNFα, and GGT increase significantly, the dynamics of INS increase and GLU and NEFA decrease is less pronounced, and the dynamics of Ca, CHOL, and TGC decrease are more evident during the experimental period compared to cows in the thermoneutral period. These findings indicate a reduction in glucose oxidation and a weakened antilipolytic effect with reduced liver functionality. Therefore, it can be concluded that in cows in the first month of lactation, the homeoretic metabolic stress caused by lactation and heat stress are opposed. Maintaining homeoretic metabolic stress in early lactation preserves milk production, so, in cows during this period, milk production decreases significantly less compared to cows in mid-lactation, when observing the entire lactation curve [67]. Maintaining lactation and homeoretic metabolic processes increases the heat production in cows in early lactation. Therefore, in these cows, a faster increase in body temperature and respiration occurs along with a decrease in the THI threshold when the presence of heat stress can be confirmed [7]. The mentioned decrease in the THI threshold is greater, i.e., heat stress occurs earlier, if the cows produce more milk [7]. The same authors found that, in cows in early lactation, the THI threshold above which heat stress occurs is lower, considering the aspect of the maximum temperature measured in a region. However, if the average temperature of the region was used, then the lactation period had no significant effect.

We determined the correlation between the examined blood parameters and the temperature of the surface of different regions of the body. The results of this study indicate that the greatest correlation was established between eHsp70, TNFα, CORT, NEFA, GLU, and RQUICKI and the surface temperatures of almost all regions of the body. Rectal temperature and body surface temperature showed a positive correlation with pro-inflammatory indicators such as TNFα and eHsp70. TNFα has peripheral and central effects on the development of thermogenesis and fever, so this finding is logical. It is thought that Hsp70, stimulated during cold stress, is useful for raising thermogenesis, and when it occurs in the extracellular space it shows pro-inflammatory effects, which probably raises the body temperatures of cows under heat stress. Hsp70 will be elevated in the circulation either due to increased cell death or the increased production that typically occurs in heat stress. During heat stress, the concentration of CORT increases in parallel with the increase in body surface temperature, probably due to glucocorticoid-induced thermogenesis. However, two other hormones important in the body’s thermogenesis process, T3 and T4, negatively correlate with the body surface temperature. The reason is probably that cortisol is of great importance in the gluconeogenesis in cows, and glucose is needed to replace fat for energy purposes in order to reduce heat production. The body surface temperature is related to metabolic flows that aim to reduce the caloric yield of metabolic reactions in heat-stress homeoresis, so a positive correlation was found with glucose, insulin, and insulin sensitivity, and a negative correlation with NEFA. With a rise in temperature, fatty acids are used less for energy purposes, which is a form of body protection. Metabolic indicators of proteolysis are a decrease in protein values and an increase in urea and creatinine values. As the values of ALB and TPROT decrease and the values of urea and Creat increase, the surface temperature of the body increases. Endocrine and metabolic changes correlate well with surface temperatures but not with rectal temperature. The main cooling process in heat stress is evaporation through the skin. In order for cooling to be possible, it is necessary that the temperature of the body’s skin (surface temperature) be as low as possible in order to enable the transfer of heat from the body’s core. However, if the temperature of the body’s skin is higher, then the heat transfer process from the core is reduced and heating occurs with the development of a metabolic response. It is possible that the blocking of heat release is of much greater importance for metabolic adaptation than increased thermogenesis in the body core, so the correlation of skin surface temperature with metabolic parameters was much more significant.

The strongest correlations with metabolic parameters were exhibited by the temperatures of the entire head, the entire body, and the abdomen, compared to the other regions. In previous research, infrared thermography in cows was used to identify the inflammation in mastitis, lameness, foot and mouth disease, and a febrile response in bovine viral diarrhea and bovine respiratory disease, and in the assessment of diseases in early lactation [24]. Regarding metabolic changes in humans, it has been demonstrated that infrared body temperature correlates with glycolyzed hemoglobin as an indicator of insulin resistance and diabetes [68]. In an experiment in cattle, a connection between infrared body temperature, creatine kinase, glucose, non-esterified fatty acids, and magnesium was established in conditions of transport stress [69]. This suggests that non-invasive infrared thermoimaging may be used to evaluate the metabolic status of stressed cattle.

## 5. Conclusions

Heat stress leads to the development of a specific metabolic, endocrine, and inflammatory response in early and mid-lactation cows. These responses differ in early and mid-lactation in part because in early lactation there are opposing metabolic fluxes induced by lactation and heat stress. The infrared thermometry of cows under heat stress is a suitable non-invasive method for assessing heat stress. The infrared temperature of a large surface, such as the left lateral surface of the entire body, the frontal surface of the entire head, or the abdomen surface, represents a good thermal window due to its high correlation with inflammatory, endocrine, and metabolic parameters in cows during heat stress. Measuring the surface temperature of the whole body or head can be a useful tool in evaluating the metabolic response of cows because it shows an association with inflammation (TNFα, eHsp70), endocrine responses (CORT, T3, T4), an increased use of glucose and decreased use of lipids for energy purposes (INS, NEFA, GLU, RQUICKI), and protein catabolism (ALB, TPROT, urea, Creat), which underlie the thermolysis and thermogenesis in cows under heat stress. In future research, it will be necessary to examine the causality between the body surface area and metabolic parameters.

## Figures and Tables

**Table 1:** Influence of heat stress and lactation period on body surface temperature.

Body Temperature (°C)	Thermoneutral Period		Mild Heat Stress		Moderate to Severe Heat Stress		Heat Stress Period	Lactation Period	Heat Stress × Lactation Period
	Early Lactation	Mid-Lactation	Early Lactation	Mid-Lactation	Early Lactation	Mid-Lactation			
Rectal	38.5 ± 0.38 ^a^	38.4 ± 0.42 ^a^	38.9 ± 0.51 ^b^	38.9 ± 0.56 ^b^	39.3 ± 0.48 ^c^	39.4 ± 0.49 ^c^	0.018	0.59	0.48
Eye	27.8 ± 0.42 ^a^	28.1 ± 0.41 ^a^	33.0 ± 0.36 ^b^	33.2 ± 0.38 ^b^	33.9 ± 0.39 ^c^	34.1 ± 0.40 ^c^	0.0013	0.49	0.36
Ear	18.6 ± 0.65 ^a^	18.9 ± 0.63 ^a^	30.4 ± 0.69 ^b^	30.6 ± 0.56 ^b^	32.0 ± 0.34 ^c^	32.2 ± 0.35 ^c^	0.0001	0.23	0.26
Nose	28.9 ± 0.32 ^a^	28.7 ± 0.31 ^a^	31.2 ± 0.35 ^b^	31.4 ± 0.37 ^b^	32.3 ± 0.30 ^c^	32.4 ± 0.34 ^c^	0.0015	0.19	0.58
Forehead	23.1 ± 0.38 ^a^	23.4 ± 0.41 ^a^	29.1 ± 0.59 ^b^	29.4 ± 0.57 ^b^	30.2 ± 0.50 ^c^	30.3 ± 0.57 ^c^	0.0021	0.31	0.32
Whole head	23.2 ± 0.95 ^a^	23.5 ± 0.99 ^a^	30.6 ± 0.78 ^b^	30.9 ± 0.81 ^b^	31.4 ± 0.86 ^c^	31.6 ± 0.79 ^c^	0.0002	0.52	0.37
Abdomen	28.5 ± 0.61 ^a^	28.6 ± 0.60 ^a^	35.9 ± 0.70 ^b^	36.1 ± 0.68 ^b^	37.8 ± 0.32 ^c^	37.9 ± 0.32 ^c^	0.0001	0.56	0.42
Udder	31.5 ± 0.44 ^a^	31.3 ± 0.42 ^a^	36.1 ± 0.51 ^b^	35.8 ± 0.49 ^b^	38.5 ± 0.27 ^c^	38.4 ± 0.25 ^c^	0.0003	0.19	0.13
Front limb	27.9 ± 0.42 ^a^	27.9 ± 0.31 ^a^	33.8 ± 0.63 ^b^	33.9 ± 0.59 ^b^	36.8 ± 0.42 ^c^	36.9 ± 0.41 ^c^	0.0002	0.28	0.15
Hind limb	27.7 ± 0.43 ^a^	27.5 ± 0.50 ^a^	33.5 ± 0.59 ^b^	33.7 ± 0.48 ^b^	37.2 ± 0.60 ^c^	37.4 ± 0.58 ^c^	0.0001	0.31	0.19
Whole body	28.8 ± 1.21 ^a^	28.9 ± 1.11 ^a^	35.7 ± 1.13 ^b^	35.9 ± 1.17 ^b^	37.7 ± 0.56 ^c^	37.6 ± 0.62 ^c^	0.0001	0.62	0.28

^a,b,c,^—different superscripts mean significant differences between groups.

**Table 2:** Influence of heat stress, lactation period, and their interaction on blood metabolic parameters.

Blood Parameters	Thermoneutral Period		Mild Heat Stress		Moderate to Severe Heat Stress		Heat Stress Period	Lactation Period	Heat Stress × Lactation Period
	Early Lactation	Mid-Lactation	Early Lactation	Mid-Lactation	Early Lactation	Mid-Lactation			
eHsp70(ng/mL) *	3.99 ± 0.65 ^a^	3.14 ± 0.78 ^b^	7.69 ± 1.15 ^c^	5.11 ± 0.61 ^d^	10.13 ± 1.03 ^e^	6.32 ± 0.95 ^c^	0.001	0.005	0.005
TNFα(ng/mL)	7.55 ± 2.1 ^a^	5.02 ± 1.4 ^b^	10.14 ± 2.1 ^c^	4.03 ± 1.62 ^d^	12.5 ± 1.98 ^e^	8.07 ± 1.2 ^f^	0.001	0.005	0.005
CORT (nmol/L)	19.9 ± 5.1 ^a^	18.1 ± 4.2 ^a^	26.5 ± 3.9 ^b^	22.3 ± 4.2 ^b^	25.6 ± 3.5 ^b^	23.1 ± 3.4 ^b^	0.001	0.751	0.624
T3 (nmol/L)	1.02 ± 0.15 ^a^	1.13 ± 0.16 ^a^	0.84 ± 0.11 ^b^	0.93 ± 0.12 ^b^	0.67 ± 0.15 ^c^	0.70 ± 0.14 ^c^	0.001	0.382	0.412
T4 (nmol/L)	36.3 ± 5.2 ^a^	41.5 ± 6.9 ^a^	32.2 ± 4.2 ^b^	36.7 ± 4.8 ^b^	25.5 ± 3.9 ^c^	26.9 ± 4.07 ^c^	0.001	0.561	0.513
INS (mU/L)	5.55 ± 1.1 ^a^	7.03 ± 0.98 ^b^	7.21 ± 0.98 ^b^	8.14 ± 1.25 ^c^	6.11 ± 1.33 ^b^	9.12 ± 1.41 ^c^	0.009	0.048	0.046
NEFA(mmol/L)	0.75 ± 0.08 ^a^	0.55 ± 0.7 ^b^	0.49 ± 0.07 ^b^	0.46 ± 0.08 ^b^	0.62 ± 0.05 ^c^	0.38 ± 0.07 ^d^	0.001	0.005	0.005
GLU (mmol/L)	3.14 ± 0.21 ^a^	3.51 ± 0.28 ^b^	2.24 ± 0.21 ^c^	2.59 ± 0.23 ^d^	2.01 ± 0.27 ^e^	2.31 ± 0.20 ^c^	0.001	0.045	0.009
RQUICKI	0.41 ± 0.05 ^a^	0.42 ± 0.04 ^b^	0.47 ± 0.04 ^c^	0.45 ± 0.04 ^d^	0.47 ± 0.05 ^c^	0.46 ± 0.05 ^e^	0.001	0.012	0.007
BHB (mmol/L)	0.75 ± 0.10 ^a^	0.63 ± 0.09 ^b^	0.54 ± 0.08 ^c^	0.49 ± 0.07 ^c^	0.81 ± 0.09 ^d^	0.70 ± 0.08 ^d^	0.008	0.314	0.43
Ca (mmol/L)	2.47 ± 0.09 ^a^	2.55 ± 0.11 ^b^	2.31 ± 0.10 ^c^	2.42 ± 0.09 ^d^	2.19 ± 0.08 ^e^	2.35 ± 0.07 ^c^	0.001	0.005	0.005
P (mmol/L)	2.99 ± 0.25 ^a^	2.81 ± 0.21 ^a^	2.45 ± 0.19 ^b^	2.41 ± 0.21 ^b^	2.29 ± 0.18 ^c^	2.30 ± 0.16 ^c^	0.001	0.822	0.752
TPROT (g/L)	63.21 ± 6.5 ^a^	68.2 ± 5.2 ^a^	55.4 ± 4.61 ^b^	53.2 ± 5.02 ^b^	52.5 ± 4.33 ^c^	51.8 ± 3.28 ^c^	0.001	0.692	0.513
ALB (g/L)	35.4 ± 2.99 ^a^	38.6 ± 2.86 ^a^	30.5 ± 2.41 ^b^	33.6 ± 2.43 ^b^	32.5 ± 2.39 ^c^	36.1 ± 2.41 ^c^	0.001	0.683	0.413
Urea (mmol/L)	4.71 ± 0.78 ^a^	4.29 ± 0.71 ^a^	5.92 ± 0.89 ^b^	4.94 ± 0.91 ^a^	6.31 ± 0.95 ^b^	5.11 ± 0.78 ^a^	0.045	0.324	0.294
Creat (µmol/L)	85.5 ± 6.3 ^a^	91.5 ± 6.5 ^a^	106.2 ± 7.2 ^b^	101.4 ± 7.3 ^b^	118.6 ± 7.5 ^c^	116.8 ± 6.9 ^c^	0.045	0.214	0.352
TGC (mmol/L)	0.28 ± 0.04 ^a^	0.35 ± 0.03 ^b^	0.21 ± 0.03 ^c^	0.28 ± 0.04 ^a^	0.15 ± 0.04 ^d^	0.28 ± 0.04 ^a^	0.007	0.043	0.048
CHOL (mmol/L)	3.73 ± 0.29 ^a^	4.21 ± 0.30 ^b^	3.42 ± 0.29 ^c^	3.96 ± 0.25 ^d^	3.01 ± 0.21 ^e^	3.9 ± 0.27 ^d^	0.008	0.046	0.045
TBIL (µmol/L)	5.91 ± 1.13 ^a^	4.22 ± 1.11 ^a^	7.12 ± 1.21 ^b^	6.99 ± 1.14 ^b^	8.03 ± 1.56 ^c^	7.86 ± 1.57 ^b^	0.009	0.761	0.672
AST (U/L)	86.5 ± 9.53 ^a^	75.4 ± 8.99 ^b^	105.8 ± 10 ^c^	89.1 ± 9.45 ^a^	114.3 ± 11.2 ^d^	99.3 ± 10.6 ^c^	0.001	0.05	0.332
GGT (U/L)	27.4 ± 3.21 ^a^	23.9 ± 2.99 ^b^	36.7 ± 3.04 ^c^	28.3 ± 3.14 ^a^	40.2 ± 4.56 ^d^	30.7 ± 4.02 ^e^	0.001	0.005	0.005
LDH (U/L)	789 ± 40 ^a^	756 ± 65 ^a^	1235 ± 50 ^b^	1199 ± 52 ^b^	1211 ± 53 ^c^	1204 ± 42 ^c^	0.046	0.622	0.421
ALP (U/L)	84.5 ± 15.2 ^a^	98.6 ± 13.5 ^a^	99.2 ± 14.1 ^a^	104 ± 15.3 ^a^	99.5 ± 14.5 ^a^	102 ± 12.8 ^a^	0.653	0.534	0.521
CK (U/L)	146 ± 25.3 ^a^	151 ± 21.4 ^a^	172 ± 25 ^a^	181 ± 30.4 ^a^	189 ± 30 ^a^	178 ± 32.2 ^a^	0.05	0.274	0.391

*—Abbreviations: see Section 2. ^a,b,c,d,e,f^—different superscripts mean significant differences between groups.

**Table 3:** Coefficients of correlation between body surface temperature (°C) and blood metabolic, endocrine, and inflammatory parameters.

	Rectal Temp.	Eye Temp.	Ear Temp.	Nose Temp.	Forehead Temp.	Whole Head t.	Abdomen Temp.	Udder Temp.	Front Limb t.	Hind Limb t.	Whole Body t.
eHsp70 ^a^	0.36 **^F^	0.85 ***^S^	0.86 ***^S^	0.81 ***^S^	0.89 ***^S^	0.95 ***^S^	0.93 ***^S^	0.9 ***^S^	0.81 ***^S^	0.79 ***^M^	0.96 ***^S^
TNFα	0.38 **^F^	0.69 ***^M^	0.61 ***^M^	0.74 ***^M^	0.75 ***^M^	0.84 ***^S^	0.89 ***^S^	0.93 ***^S^	0.63 ***^M^	0.65 ***^M^	0.84 ***^S^
CORT	0.16 ^P^	0.56 ***^F^	0.58 ***^F^	0.59 ***^F^	0.63 ***^M^	0.7 ***^M^	0.65 ***^M^	0.49 ***^F^	0.52 ***^F^	0.32 **^F^	0.72 ***^M^
T3	0.12 ^P^	−0.51 **^F^	−0.48 **^F^	−0.39 **^F^	−0.51 **^F^	−0.62 ***^M^	−0.6 ***^M^	−0.44 **^F^	−0.36 **^F^	−0.32 **^F^	−0.51 **^F^
T4	0.15 ^P^	−0.48 **^F^	−0.45 **^F^	−0.43 **^F^	−0.42 **^F^	−0.7 ***^M^	−0.61 ***^M^	−0.51 **^F^	−0.38 **^F^	−0.19 ^P^	−0.45 **^F^
INS	0.16 ^P^	0.61 ***^M^	0.52 **^F^	0.59 **^F^	0.63 ***^M^	0.78 ***^M^	0.8 ***^S^	0.49 **^F^	0.32 **^F^	0.22 *^P^	0.82 ***^S^
NEFA	0.12 ^P^	−0.53 **^F^	−0.49 **^F^	−0.5 **^F^	−0.52 **^F^	−0.68 ***^M^	−0.7 ***^M^	−0.52 **^F^	−0.41 **^F^	−0.36 **^F^	−0.65 ***^M^
GLU	0.11 ^P^	−0.62 ***^M^	−0.55 **^F^	−0.58 **^F^	−0.61 ***^M^	−0.8 ***^S^	−0.74 ***^M^	−0.61 ***^M^	−0.38 **^F^	−0.33 **^F^	−0.79 ***^M^
RQUICKI	0.03 ^P^	0.51 **^F^	0.59 **^F^	0.39 **^F^	0.42 **^F^	0.58 **^F^	0.49 **^F^	0.35 **^F^	0.42 **^F^	0.29 **^P^	0.83 ***^S^
BHB	0.06 ^P^	−0.26 *^P^	−0.18 ^P^	−0.23 *^P^	−0.29 **^P^	−0.3 **^F^	−0.39 **^F^	−0.3 **^F^	−0.25 *^P^	−0.19 ^P^	−0.57 **^F^
Ca	0.02 ^P^	−0.15 ^P^	−0.2 ^P^	−0.19 ^P^	−0.16 ^P^	−0.25 *^P^	−0.3 **^F^	−0.15 ^P^	−0.11 ^P^	−0.1 ^P^	−0.51 **^F^
P	0.03 ^P^	−0.1 ^P^	−0.09 ^N^	−0.08 ^N^	−0.1 ^P^	−0.16 ^P^	−0.2 *^P^	−0.08 ^N^	−0.02 ^N^	−0.02 ^N^	−0.29 **^P^
TPROT	0.05 ^P^	−0.34 **^F^	−0.31 **^F^	−0.35 **^F^	−0.41 **^F^	−0.46 **^F^	−0.4 **^F^	−0.33 **^F^	−0.21 *^P^	−0.18 ^P^	−0.53 **^F^
ALB	0.07 ^P^	−0.21 *^P^	−0.2 ^P^	−0.19 ^P^	−0.25 *^P^	−0.39 **^F^	−0.3 **^F^	−0.25 *^P^	−0.18 ^P^	−0.15 ^P^	−0.37 **^F^
Urea	0.14 ^P^	0.48 **^F^	0.45 **^F^	0.43 **^F^	0.5 **^F^	0.57 ***^F^	0.58 **^F^	0.39 **^F^	0.4 **^F^	0.42 **^F^	0.62 ***^M^
Creat	0.03 ^P^	0.59 **^F^	0.55 **^F^	0.58 **^F^	0.61 ***^M^	0.7 ***^M^	0.78 ***^M^	0.68 ***^M^	0.59 **^F^	0.55 **^F^	0.81 ***^S^
TGC	0.05 ^P^	−0.42 **^F^	−0.45 **^F^	−0.41 **^F^	−0.55 **^F^	−0.55 **^F^	−0.57 **^F^	−0.49 **^F^	−0.32 **^F^	−0.28 **^F^	−0.64 **^M^
CHOL	0.08 ^P^	−0.35 **^F^	−0.36 **^F^	−0.38 **^F^	−0.39 **^F^	−0.4 **^F^	−0.45 **^F^	−0.5 **^F^	−0.32 **^F^	−0.29 **^F^	−0.55 **^F^
TBIL	0.06 ^P^	0.21 *^P^	0.21 *^P^	0.19 ^P^	0.15 ^P^	0.21 *^P^	0.36 **^F^	0.38 **^F^	0.18 ^P^	0.17 ^P^	0.36 **^F^
AST	0.13 ^P^	0.39 **^F^	0.42 **^F^	0.45 **^F^	0.4 **^F^	0.48 **^F^	0.52 **^F^	0.65 ***^M^	0.47 **^F^	0.39 **^F^	0.72 ***^M^
GGT	0.09 ^P^	0.4 **^F^	0.38 **^F^	0.36 **^F^	0.39 **^F^	0.45 **^F^	0.52 **^F^	0.3 **^F^	0.25 *^P^	0.19 ^P^	0.69 ***^M^
LDH	0.11 ^P^	0.23 *^P^	0.25 *^P^	0.28 **^P^	0.35 **^F^	0.29 **^P^	0.41 **^F^	0.48 **^F^	0.32 **^F^	0.25 *^P^	0.63 ***^M^
ALP	0.09 ^P^	0.08 ^N^	0.06 ^N^	0.05 ^N^	0.12 ^P^	0.15 ^P^	0.2 ^P^	0.21 *^P^	0.07 ^N^	0.03 ^F^	0.15 ^P^
CK	0.04 ^P^	0.11 ^P^	0.15 ^P^	0.21 *^P^	0.15 ^P^	0.23 *^P^	0.21 *^P^	0.18 ^P^	0.25 *^P^	0.26 *^P^	0.31 **^F^

^a^—Abbreviations: see Section 2. Units are the same as in Table 2; * *p* < 0.05; ** *p* < 0.01; *** *p* < 0.001; strength of the correlation was assessed based on the correlation coefficient as no (N, r = 0.00–0.09), poor (P, r = 0.1–0.29), fair (F, r = 0.3–0.59), moderate (M, r = 0.6–0.79), strong (S, r = 0.8–0.99), or perfect (Pe, r = 1) correlation.

## Data Availability

The data presented in this study are available within the article.

## References

[1] Cincović M.R., Majkić M., Belić B., Plavša N., Lakić I., Radinović M. (2017). Thermal comfort of cows and temperature humidity index in period of 2005–2016 in Vojvodina region (Serbia). Acta Agric. Serbica.

[2] Pecelj M.M., Lukić M.Z., Filipović D.J., Protić B.M., Bogdanović U.M. (2020). Analysis of the Universal Thermal Climate Index during heat waves in Serbia. Nat. Hazards Earth Syst. Sci..

[3] Vranić P., Milutinović S. (2016). From local sustainable development towards climate change adaptation: A case study of Serbia. Int. J. Sustain. Dev. World Ecol..

[4] Collier R.J., Gebremedhin K.G. (2015). Thermal biology of domestic animals. Annu. Rev. Anim. Biosci..

[5] Liu J., Li L., Chen X., Lu Y., Wang D. (2019). Effects of heat stress on body temperature, milk production, and reproduction in dairy cows: A novel idea for monitoring and evaluation of heat stress—A review. Asian-Australas. J. Anim. Sci..

[6] Cincovic M.R., Belic B., Toholj B., Potkonjak A., Stevancevic M., Lako B., Radovic I. (2011). Metabolic acclimation to heat stress in farm housed Holstein cows with different body condition scores. Afr. J. Biotechnol..

[7] Yan G., Liu K., Hao Z., Shi Z., Li H. (2021). The effects of cow-related factors on rectal temperature, respiration rate, and temperature-humidity index thresholds for lactating cows exposed to heat stress. J. Therm. Biol..

[8] Toledo I.M., Fabris T.F., Tao S., Dahl G.E. (2020). When do dry cows get heat stressed? Correlations of rectal temperature, respiration rate, and performance. JDS Commun..

[9] Wijffels G., Sullivan M., Gaughan J. (2021). Methods to quantify heat stress in ruminants: Current status and future prospects. Methods.

[10] Mota-Rojas D., Pereira A.M.F., Wang D., Martínez-Burnes J., Ghezzi M., Hernández-Avalos I., Lendez P., Mora-Medina P., Casas A., Olmos-Hernández A. (2021). Clinical Applications and Factors Involved in Validating Thermal Windows Used in Infrared Thermography in Cattle and River Buffalo to Assess Health and Productivity. Animals.

[11] Zhou M., Koerkamp P.G., Huynh T.T.T., Aarnink A.J.A. (2023). Evaporative water loss from dairy cows in climate-controlled respiration chambers. J. Dairy Sci..

[12] Hoffmann G., Herbut P., Pinto S., Heinicke J., Kuhla B., Amon T. (2020). Animal-related, non-invasive indicators for determining heat stress in dairy cows. Biosyst. Eng..

[13] Wheelock J.B., Rhoads R.P., VanBaale M.J., Sanders S.R., Baumgard L.H. (2010). Effects of heat stress on energetic metabolism in lactating Holstein cows. J. Dairy Sci..

[14] Farooq U., Samad H.A., Shehzad F., Qayyum A. (2010). Physiological responses of cattle to heat stress. World Appl. Sci. J..

[15] Majkić M., Cincović M.R., Belić B., Plavša N., Lakić I., Radinović M. (2017). Relationship between milk production and metabolic adaptation in dairy cows during heat stress. Acta Agric. Serbica.

[16] Tejaswi V., Balamurugan B., Samad H.A., Sarkar M., Maurya V.P., Singh G. (2020). Differential endocrine and antioxidant responses to heat stress among native and crossbred cattle. J. Vet. Behav..

[17] Kim W.S., Ghassemi Nejad J., Roh S.G., Lee H.G. (2020). Heat-shock proteins gene expression in peripheral blood mononuclear cells as an indicator of heat stress in beef calves. Animals.

[18] Krause M., Heck T.G., Bittencourt A., Scomazzon S.P., Newsholme P., Curi R., Homem de Bittencourt P.I. (2015). The chaperone balance hypothesis: The importance of the extracellular to intracellular HSP70 ratio to inflammation-driven type 2 diabetes, the effect of exercise, and the implications for clinical management. Mediat. Inflamm..

[19] Ruiz-González A., Rico D.E., Rico J.E. (2022). Modulation of fecal metabolites by heat stress and diet, and their association with inflammation and leaky gut markers in dairy cows. Metabolites.

[20] Mezzetti M., Cattaneo L., Passamonti M.M., Lopreiato V., Minuti A., Trevisi E. (2021). The transition period updated: A review of the new insights into the adaptation of dairy cows to the new lactation. Dairy.

[21] Kendall P.E., Webster J.R. (2009). Season and physiological status affects the circadian body temperature rhythm of dairy cows. Livest. Sci..

[22] Sammad A., Wang Y.J., Umer S., Lirong H., Khan I., Khan A., Ahmad B., Wang Y. (2020). Nutritional Physiology and Biochemistry of Dairy Cattle under the Influence of Heat Stress: Consequences and Opportunities. Animals.

[23] Godyń D., Herbut P., Angrecka S. (2019). Measurements of peripheral and deep body temperature in cattle—A review. J. Therm. Biol..

[24] Macmillan K., Colazo M.G., Cook N.J. (2019). Evaluation of infrared thermography compared to rectal temperature to identify illness in early postpartum dairy cows. Res. Vet. Sci..

[25] Čukić A., Rakonjac S., Djoković R., Cincović M., Bogosavljević-Bošković S., Petrović M., Savić Ž., Andjušić L., Andjelić B. (2023). Influence of Heat Stress on Body Temperatures Measured by Infrared Thermography, Blood Metabolic Parameters and Its Correlation in Sheep. Metabolites.

[26] National Research Council (2001). Nutrient Requirements of Dairy Cattle: 2001.

[27] National Research Council (1971). A Guide to Environmental Research on Animals.

[28] Cincović M.R., Belić B., Djoković R., Ježek J., Petrović M.D., Božić A., Anderson R.C., Starič J. (2019). Revised quantitative insulin sensitivity check index: Associations with the metabolic status of cows during early lactation. Vet. Arh..

[29] Armstrong D.V. (1994). Heat stress interaction with shade and cooling. J. Dairy Sci..

[30] Ravagnolo O., Misztal I., Hoogenboom G. (2000). Genetic component of heat stress in dairy cattle, development of heat index function. J. Dairy Sci..

[31] Aguilar I., Misztal I., Tsuruta S. (2010). Short communication: Genetic trends of milk yield under heat stress for US holsteins. J. Dairy Sci..

[32] Bohmanova J., Misztal I., Cole J.B. (2007). Temperature-humidity indices as indicators of milk production losses due to heat stress. J. Dairy Sci..

[33] Mbuthia J.M., Eggert A., Reinsch N. (2022). Comparison of high resolution observational and grid-interpolated weather data and application to thermal stress on herd average milk production traits in a temperate environment. Agric. For. Meteorol..

[34] Mbuthia J.M., Mayer M., Reinsch N. (2021). Modeling heat stress effects on dairy cattle milk production in a tropical environment using test-day records and random regression models. Animal.

[35] Spasojević J., Majkić M., Cincović M., Stanojević J., Blond B., Radinović M. (2023). Correlation of body surface temperature measured by infrared thermography and air temperature and humidity index (THI) in the assessment of heat stress in cows. Ann. Agron.-Letop. Naučnih Rad..

[36] Yan G., Shi Z., Li H. (2021). Critical temperature-humidity index thresholds based on surface temperature for lactating dairy cows in a temperate climate. Agriculture.

[37] Unruh E.M., Theurer M.E., White B.J., Larson R.L., Drouillard J.S., Schrag N. (2017). Evaluation of infrared thermography as a diagnostic tool to predict heat stress events in feedlot cattle. Am. J. Vet. Res..

[38] Sejian V., Shashank C.G., Silpa M.V., Madhusoodan A.P., Devaraj C., Koenig S. (2022). Non-Invasive Methods of Quantifying Heat Stress Response in Farm Animals with Special Reference to Dairy Cattle. Atmosphere.

[39] Salles M.S.V., da Silva S.C., Salles F.A., Roma L.C., El Faro L., Mac Lean P.A.B., de Oliveira C.E.L., Martello L.S. (2016). Mapping the body surface temperature of cattle by infrared thermography. J. Therm. Biol..

[40] Martello L.S., Da Luz S.S., Gomes R.C., Corte R.S.R.P., Leme P.R. (2016). Infrared thermography as a tool to evaluate body surface temperature and its relationship with feed efficiency in Bos indicus cattle in tropical conditions. Int. J. Biometeorol..

[41] Reece W.O., Erickson H.H., Goff J.P., Uemura E.E. (2015). Dukes’ Physiology of Domestic Animals.

[42] Okada K., Takemura K., Sato S. (2013). Investigation of various essential factors for optimum infrared thermography. J. Vet. Med. Sci..

[43] Montanholi Y.R., Nicholas E.O., Kendall C.S., Schenkel F.S., Mcbride B.W., Miller S.P. (2008). Application of infrared thermography as an indicator of heat and methane production and its use in the study of skin temperature in response to physiological events in dairy cattle (*Bos taurus*). J. Therm. Biol..

[44] Berry R.J., Kennedy A.D., Scott S.L., Kyle B.L., Shaefer A.L. (2003). Daily variation in the udder surface temperature of dairy cows measured by infrared thermography: Potential for mastitis detection. Can. J. Anim. Sci..

[45] Osei-Amponsah R., Dunshea F.R., Leury B.J., Cheng L., Cullen B., Joy A., Abhijith A., Zhang M.H., Chauhan S.S. (2020). Heat stress impacts on lactating cows grazing Australian summer pastures on an automatic Robotic Dairy. Animals.

[46] Bertoni A., Mota-Rojas D., Álvarez-Macias A., Mora-Medina P., Guerrero-Legarreta I., Morales-Canela A., Gómez-Prado J., José-Pérez N., Martínez-Burnes J. (2020). Scientific findings related to changes in vascular microcirculation using infrared thermography in the river buffalo. J. Anim. Behav. Biometeorol..

[47] Chen S., Wang J., Peng D., Li G., Chen J., Gu X. (2018). Exposure to heat-stress environment affects the physiology, circulation levels of cytokines, and microbiome in dairy cows. Sci. Rep..

[48] Alvarez M., Johnson H. (1973). Environmental Heat Exposure on Cattle Plasma Catecholamine and Glucocorticoids. J. Dairy Sci..

[49] Das R., Sailo L., Verma N., Bharti P., Saikia J. (2016). Impact of heat stress on health and performance of dairy animals: A review. Vet. World.

[50] de Lima Guimarães Yamada K., Dos Santos G.T., Damasceno J.C., de Almeida K.V., Osorio J.A., Lourenço J.C., Gurgel A.L., Dias-Silva T.P., de Araújo M.J., Ítavo L.C. (2023). Effects of heat-stress-reducing systems on blood constituents, milk production and milk quality of Holstein and Jersey cows and heifers on pasture. Trop. Anim. Health Prod..

[51] Anjali, Gururaj V.K., Sarma L., Tripathi M., Verma M.R., Verma V., Pathak M.C., Samad H.A., Maurya V.P., Chouhan V.S. (2023). Thyroid hormone dynamics of Tharparkar and Sahiwal cattle during induced heat stress. Trop. Anim. Health Prod..

[52] Hunninck L., Jackson C.R., May R., Røskaft E., Palme R., Sheriff M.J. (2020). Triiodothyronine (T3) levels fluctuate in response to ambient temperature rather than nutritional status in a wild tropical ungulate. Conserv. Physiol..

[53] Baumgard L.H., Wheelock J.B., Sanders S.R., Moore C.E., Green H.B., Waldron M.R., Rhoads R.P. (2011). Postabsorptive carbohydrate adaptations to heat stress and monensin supplementation in lactating Holstein cows. J. Dairy Sci..

[54] Baumgard L.H., Rhoads R.P. (2013). Effects of Heat Stress on Postabsorptive Metabolism and Energetics. Annu. Rev. Anim. Biosci..

[55] Majkić M., Cincović M., Spasojević J., Jožef I., Blond B., Kovačević D. (2023). Resilience curve and cumulative response of cows to heat stress. Ann. Agron. Letop. Naučnih Rad..

[56] Berian S., Gupta S.K., Sharma S., Ganai I., Dua S., Sharma N. (2019). Effect of heat stress on physiological and hemato-biochemical profile of cross bred dairy cattle. J. Anim. Res..

[57] Gao S.T., Guo J., Quan S.Y., Nan X.M., Fernandez M.S., Baumgard L.H., Bu D.P. (2017). The effects of heat stress on protein metabolism in lactating Holstein cows. J. Dairy Sci..

[58] Rhoads R.P., La Noce A.J., Wheelock J.B., Baumgard L.H. (2011). Short communication: Alterations in expression of gluconeogenic genes during heat stress and exogenous bovine somatotropin administration. J. Dairy Sci..

[59] Abbas Z., Sammad A., Hu L., Fang H., Xu Q., Wang Y. (2020). Glucose Metabolism and Dynamics of Facilitative Glucose Transporters (GLUTs) under the Influence of Heat Stress in Dairy Cattle. Metabolites.

[60] Mezzetti M., Bionaz M., Trevisi E. (2020). Interaction between inflammation and metabolism in periparturient dairy cows. J. Anim. Sci..

[61] Joo S.S., Lee S.J., Park D.S., Kim D.H., Gu B.-H., Park Y.J., Rim C.Y., Kim M., Kim E.T. (2021). Changes in Blood Metabolites and Immune Cells in Holstein and Jersey Dairy Cows by Heat Stress. Animals.

[62] Cincović M.R., Djoković R., Belić B., Potkonjak A., Toholj B., Stojanac N., Stevančević O., Starič J. (2017). Inorganic phosphorus decrease after intravenous glucose tolerance test is associated with insulin resistance in dairy cows. Vet. Arh..

[63] Pascottini O.B., Leroy J.L.M.R., Opsomer G. (2020). Metabolic Stress in the Transition Period of Dairy Cows: Focusing on the Prepartum Period. Animals.

[64] Krnjaić S., Cincović M., Djoković R., Belić B., Ježek J., Starič J. (2022). The Influence of Energy Balance, Lipolysis and Ketogenesis on Metabolic Adaptation in Cows Milked Twice and Three Times Daily. Metabolites.

[65] Petrović M.Ž., Cincović M., Starič J., Djoković R., Belić B., Radinović M., Majkić M., Ilić Z.Ž. (2022). The Correlation between Extracellular Heat Shock Protein 70 and Lipid Metabolism in a Ruminant Model. Metabolites.

[66] Turk R., Rošić N., Vince S., Perkov S., Samardžija M., Beer-Ljubić B., Belić M., Robić M. (2020). The influence of heat stress on energy metabolism in Simmental dairy cows during the periparturient period. Vet. Arh..

[67] Cincović M.R., Belić B.M., Toholj B.D., Radović I.V., Vidović B.R. (2010). The influence of THI values at different periods of lactation on milk quality and characteristics of lactation curve. J. Agric. Sci..

[68] Sivanandam S., Anburajan M., Venkatraman B., Menaka M., Sharath D. (2012). Medical thermography: A diagnostic approach for type 2 diabetes based on non-contact infrared thermal imaging. Endocrine.

[69] Cuthbertson H., Tarr G., Loudon K., Lomax S., White P., McGreevy P., Polkinghorne R., González L.A. (2020). Using infrared thermography on farm of origin to predict meat quality and physiological response in cattle (*Bos taurus*) exposed to transport and marketing. Meat Sci..

